# The modified Ravitch approach for the management of severe anterior flail chest with bilateral sternochondral dislocations: a case report

**DOI:** 10.1186/s40792-018-0416-5

**Published:** 2018-01-19

**Authors:** Gabriel Estremera, Ellen Chieko Omi, Eduardo Smith-Singares

**Affiliations:** 1 Department of Surgery, College of Medicine, The University of Illinois at Chicago, 840 S. Wood Street, Suite 502 Clinical Sciences Building, MC 958, Chicago, IL 60612 USA; 2 Advocate Christ Medical Center, 4440 W 95th Street Suite 183 S, Oak Lawn, IL 60453 USA

**Keywords:** Flail chest, Thoracic trauma, Chest wall osteosythesis, Modified Ravitch approach, Case report

## Abstract

The management of flail chest continues to evolve as scientific evidence and surgical experience accumulates. Flail chest injuries that span the sternum present a rare and complicated injury pattern that can be challenging to manage both medically and surgically. Our patient is a 69-year-old involved in a high-speed motor vehicle crash with respiratory failure secondary to an anterior flail chest. Tomographic examination confirmed a sternal fracture with bilateral sternochondral dislocations and multiple rib fractures. The rib fractures created a lateral flail segment which extended towards the right side. An open Pectus exposure with a right anterolateral extension (modified Ravitch approach) and osteosynthesis plates accomplished stabilization of the chest wall, and contributed to weaning from mechanical ventilation.

**Electronic supplementary material:**

The online version of this article (10.1186/s40792-018-0416-5) contains supplementary material, which is available to authorized users.

## Background

Flail chest continues to present significant therapeutic challenges to both clinicians and their patients. While evidence continues to accumulate regarding the treatment of multiple, sequential rib fractures, certain combinations of chest wall instability lack specific recommendations and guidelines for their management, mostly due to their low incidence. One such presentation is the sternochondral dislocation associated with sternal body fracture.

In this article, we present the management of such an injury. The case report was written following the recommendations for the conduct, reporting, editing, and publication of scholarly work in medical journals (contained in the ICJME statement, http://www.icmje.org/), the work of the Equator Network (http://www.equator-network.org/) and the SCARE checklist [1].

## Case presentation

A 69-year-old white male with a history of chronic obstructive pulmonary disease, hypertension and rheumatoid arthritis on steroids was brought to the emergency department after a head on collision at 50 miles per hour. He was in respiratory distress with obvious chest wall deformity. Prehospital interventions included a needle decompression on the right side. On arrival, his airway was patent, with bilateral diminished breath sounds and saturating 97% on 100% nonrebreather mask. He was tachypneic with obvious paradoxical movement of the right side of his chest wall with retraction upon inspiration bilaterally. Significant subcutaneous emphysema was noted, and the abnormal chest motion extended towards the right anterior-lateral chest wall (Additional file 1: Video S1). He was normotensive with a heart rate in 90 s. His presenting GCS was 15, with no obvious neurologic deficits. There was no any evidence of abdominal or pelvic tenderness, and the remainder of his primary survey was notable only for some lower extremity bruising. Chest tubes were placed bilaterally, after the patient was intubated and sedated for persistent respiratory distress and agitation with impending respiratory collapse. Output from the right chest tube was 120 mL and from the left chest tube 120 mL. The chest tubes were placed based on the enormous amount of subcutaneous emphysema, but very small pneumothorax and hemothorax on initial chest X-ray.

**Fig. 1:**
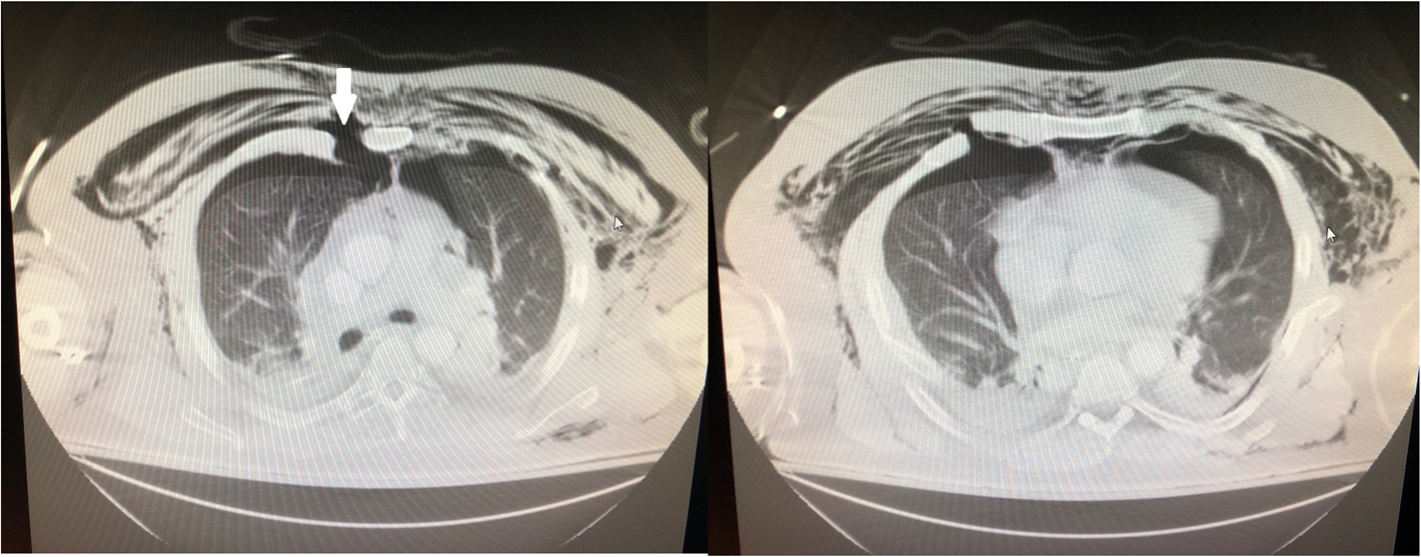
Initial chest CT (Lung Windows) axial cuts, showing extensive subcutaneous emphysema, with associated bilateral pneumothoraces, small residual bilateral hemothoraces and associated pulmonary contusions. The right panel shows a displaced 3rd rib sternochondral dislocation (solid white arrow), providing a clear path for a possible pulmonary hernia. The left panel shows displaced rib fractures just inferior to the right sternochondral dislocation, creating an anterior flail segment

**Fig. 2:**
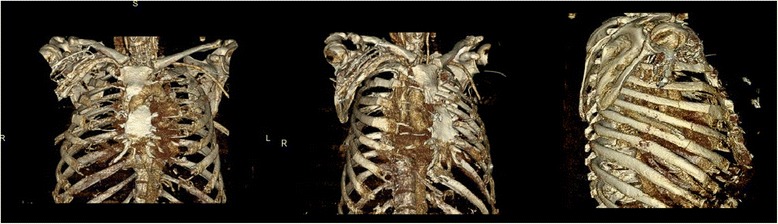
3D reconstruction of the anterior chest wall (rotational views) showing on the AP panel displaced sternal fracture, disruption of the sternochondral junctions in both the right and the left, on the oblique panel significant diastasis of the sternochondral junctions on the right side and multiple fractures of the second though sixth rib on the right side are demonstrated. Finally on the lateral panel showing a flail segment (two fractures on three consecutive ribs) between the third and the sixth rib

Chest and abdominal X-rays were obtained and showed subcutaneous emphysema and an endotracheal tube in adequate position. The patient was subsequently taken to CT scan for imaging (Fig. 1) with 3D reconstructions (Fig. 2) which revealed significant chest wall deformity with multiple fractures. The imaging demonstrated separation of the right 3rd to 5th costochondral junctions, separation of the left 2nd costochondral junction, and an oblique fracture of the proximal sternum with a 1.5-cm gap of displacement, and multiple rib fractures bilaterally. The right hemithorax had fractures of the anterior lateral 1st to 8th ribs and the left hemithorax had anterior-lateral fracture of ribs 2–6. The patient had an anatomic flail chest just from the right ribs 3–6 breaking in more than one place, but also a clinical and anatomic flail from the bilateral anterior lateral rib fractures and sternal fracture (Additional file 1: Video S1).


**Additional file 1: Video S1.** (MP4 2931 kb)


### Clinical management

**Fig. 3:**
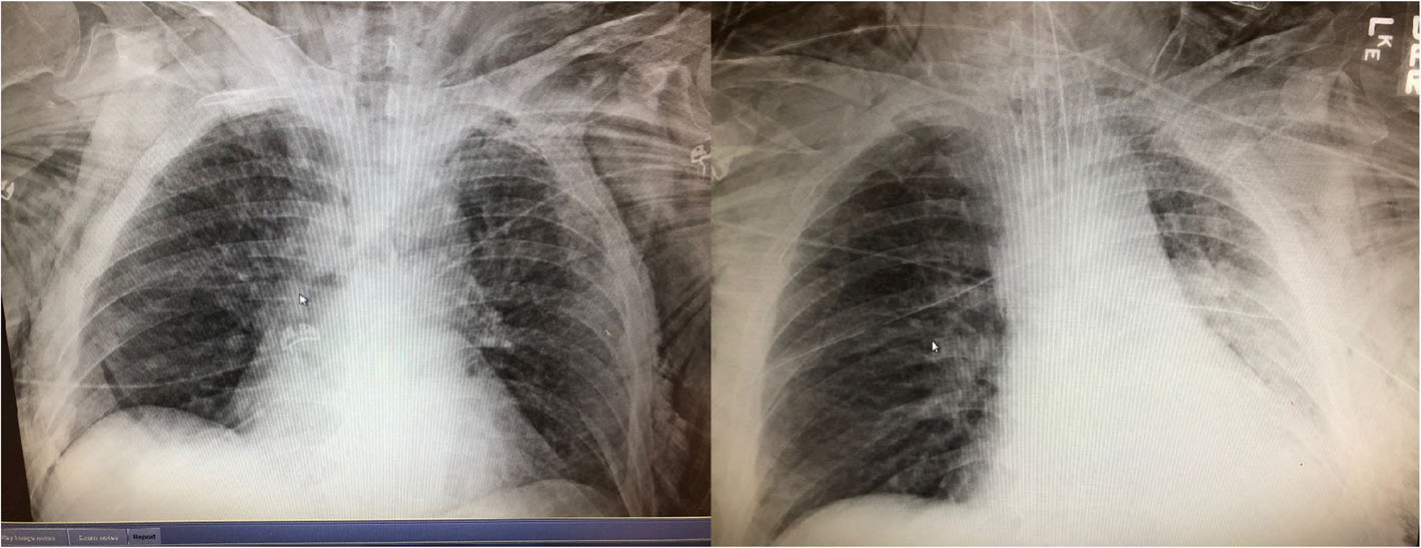
Progression of preoperative chest X-ray findings, showing an evolving large pulmonary contusion with an atelectatic component on the left pulmonary field

The patient was brought to the Surgical & Trauma Intensive Care Unit (STICU) for management of acute respiratory failure. He required vasopressors initially upon arrival to the STICU, but they were weaned off within a few hours. Over the course of several days, the patient developed significant pulmonary contusions, with increased oxygen to 60% and PEEP 10 requirements. Sedation and analgesia with continuous drips of midazolam hydrochloride (Versed, F. Hoffmann-La Roche Ltd., Basel Switzerland) and fentanyl (Sublimaze, Janssen Pharmaceutica, Raritan NJ) could not be weaned due to persistent agitation and mechanical respiratory failure from to the paradoxical movement of his anterolateral chest wall. His chest X-ray demonstrated continued worsening of opacifications of the left lung, consistent with an evolving pulmonary contusion (Fig. 3). The patient’s respiratory status was further complicated by exacerbations of asthma requiring continuous nebulizer treatment. On hospital day 5, the patient was brought to the operating room for stabilization of his flail chest and chest wall reconstruction.

### Surgical technique

**Fig. 4:**
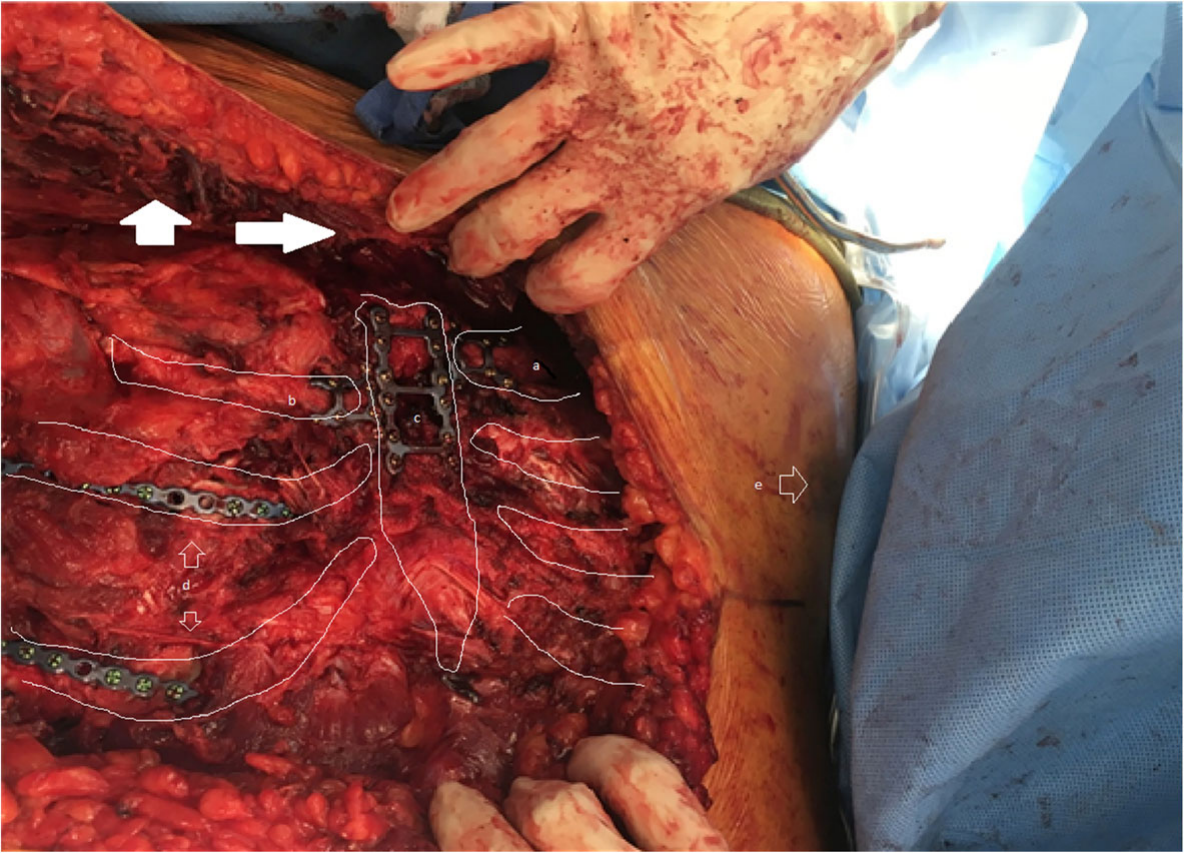
Operative view of the reconstruction, showing: (a) repair of the sternochondral dislocation of the left side, (b) repair of the sternochondral dislocation on the right side, (c) Sternal repair, (d) the flail chest repair with ribs stabilization, (e) left nipple. The solid white arrows points to the myocutaneous pectoral flap already elevated

The patient was placed on the operating table in the supine position with the arms extended out to the sides. The chest was prepped bilaterally to the posterior axillary lines. A right inframammary thoracotomy incision was made, which extended past the midline at about the level of the 6 rib space, with a cephalad extension to provide sternal exposure, and continued along the left inframammary line (modified Ravitch incision). Superior and inferior musculocutaneous flaps were created by raising the pectoralis major off the chest wall to expose the injured ribs and sternum. The flaps were extended to above the level of the manubrium superiorly and to the rectus muscle inferiorly. Once the sternum was exposed anteriorly, the bone was released posteriorly from the anterior mediastinal structures using blunt dissection. Both mammary pedicles were identified and preserved. A caliper was used to determine the length of screw necessary for bicortical fixation. The sternum was initially reduced using clamps to achieve necessary approximation. A Biomet H-plate (Zimmer Biomet, Warsaw IN) was placed over the fracture line and fixated using 18- and 20-mm screws for bicortical fixation (Fig. 4).

After approximation of the sternum was complete, the displaced ribs were stabilized using rib plates (Zimmer Biomet, Warsaw IN). The reconstruction of the ribs was accomplished with fixation of the 4th, 5th, and 6th ribs on the right side with plates that bridged both the anterior and lateral fractures. The second left and third right costochondral cartilage were also significantly displaced from the sternum, and the approximation of the joint was achieved using a Biomet LJ plate (Zimmer Biomet, Warsaw IN) bent to fit the bone and cartilage on either side.

### Follow-up and outcome

**Fig. 5:**
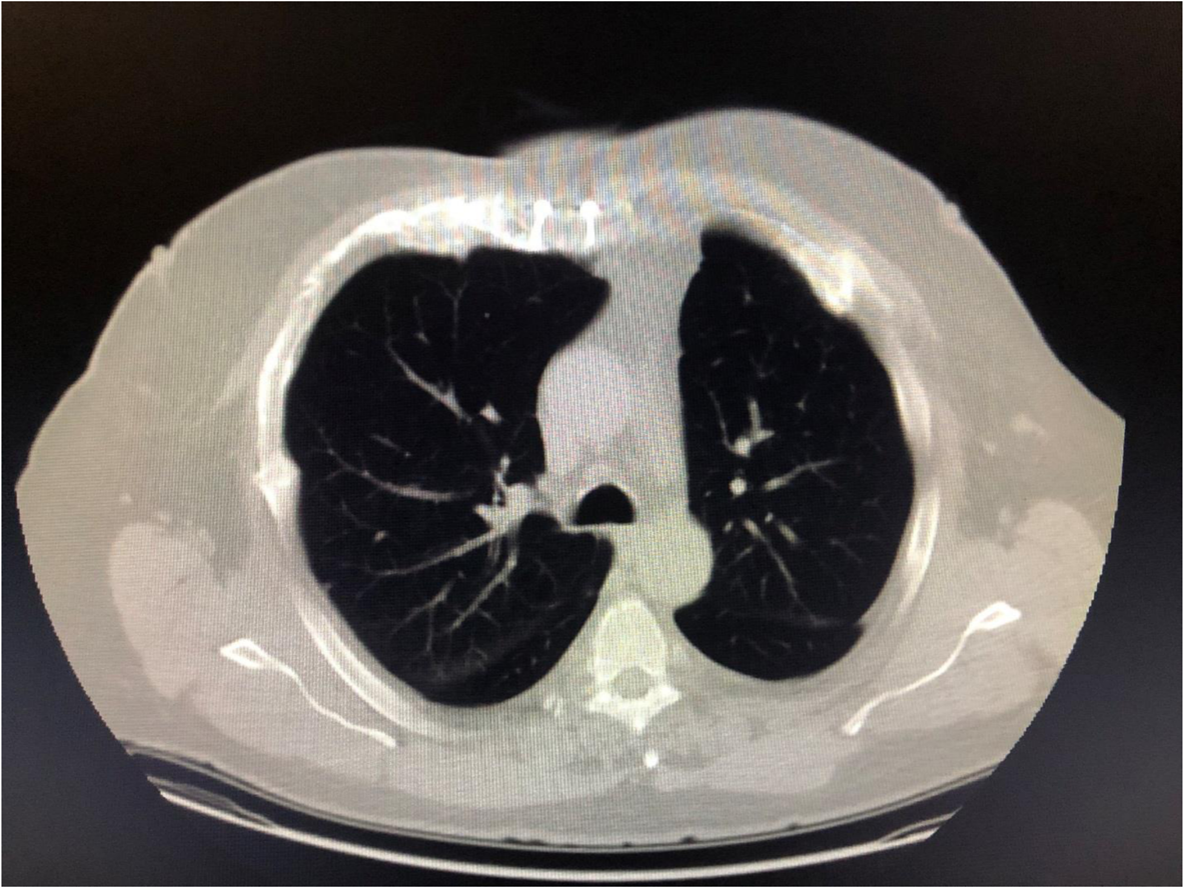
Follow-up tomography 9 months postop, showing resolution of the flail chest and hardware in place

The patient was returned to the STICU for further management postoperatively. Attempted extubation on postoperative day 3 resulted in reintubation for severe agitation after 1 h. He developed ventilator-associated pneumonia, which further complicated his weaning. He was treated and finally extubated on postoperative day 11. Additional events during his STICU stay included delirium and dysphagia, both of which resolved. He was transferred out of the STICU on postoperative day 13 and was discharged to a long-term acute care facility on postoperative day 15, on oxycodone IR (Mayne Pharma, Raleigh NC) 10 mg q4h and tramadol (Ultram, Janssen Pharmaceutica, Raritan NJ) 50 mg q4h for analgesia. The patient received follow-up at the Trauma clinic 2 weeks and 2 months after discharge. The patient returned to our trauma center at 3 months, with a sinus tract arising from the right fourth rib hardware, draining seropurulent material. Cultures of this material yielded *Enterobacter clocae*. He required outpatient antibiotic therapy for a total of 8 weeks. Antibiotics suppressed the infection, but because the infection failed to resolve completely, revision and explantation of the infected plate 5 months postoperatively was required. Since then, he has made significant progress towards his recovery. Follow-up tomography 9 months after surgery shows complete healing and resolution of the flail chest (Fig. 5).

### Discussion

Flail chest is a severe, life-threatening complication of thoracic trauma. The condition is defined as three or greater consecutive ribs fractured in two places, with or without a sternal component, with associated paradoxical motion of the chest wall segment. This affected segment is in discontinuity with the rest of the chest wall, leading to collapse on inspiration and expansion upon expiration [2]. Clinical significance of the flail segment can vary based on size and anatomic location. Morbidity associated with the condition includes mechanical ventilation, pneumonia, chest wall deformation, and chronic pain. Associated pulmonary contusions can further complicate patient conditions. A higher overall morbidity has been attributed to anterior flail segments as compared to lateral. Mortality rates vary between studies depending on associated injuries, but have been noted to range from 5 to 36% [3].

Current conservative management guidelines focus on avoidance of hypotension and hypoxia along with adequate pain control. Goal-directed fluid resuscitation should be used in the setting of hypotension, and epidural anesthesia is considered first-line treatment for pain in the setting of flail chest with pulmonary contusion. Mechanical ventilation is not used prophylactically, but should be employed in the setting of respiratory failure [4].

Although this conservative management is generally agreed upon, the role for surgical fixation in the setting of flail chest and the timing of therapy continues to evolve. Current indications for surgical fixation include (1) thoracotomy for other thoracic injuries, (2) unsuccessful weaning from mechanical ventilation, (3) severe chest wall instability, (4) persistent pain, and (5) progressive loss of pulmonary function [4, 5]. A randomized control trial by Tanaka et al. demonstrated statistically shorter periods of mechanical ventilation, ICU days, lower incidences of pneumonia, and less need for tracheostomy. Furthermore, several studies have found improvement in long-term pulmonary function tests and decrease in pain when compared to non-operative management [5, 6]. Of the abovementioned indications for surgical intervention, our patient displayed three. His chest wall instability was the cause for his severe pain induced agitation as well as a strong contributing factor in his failure to wean from mechanical ventilation. This particular clinical association has been described before [7].

The typical incisions for surgical stabilization of rib fractures involve a classic antero-lateral or posterior lateral thoracotomy or a vertical axillary thoracotomy for exposure of the fractured ribs. For decades, Kirschner wires and Judet plates were used with variable success. With the development of rib stabilization systems that used modern principles of osteosynthesis, there has been an explosion of systems of rib fixation hardware that both restore anatomical contour of the chest and provide the creation of a rigid construct for bone healing. While literature on the surgical fixation of lateral flail chest continues to accumulate, reports on the surgical approach to sternochondral disjunction are quite rare. A few case reports or small series have been published documenting the use of the Nuss procedure using a retrosternal bar for sternal stabilization [8]. Our patient’s condition was quite complex, involving a transverse displaced sternal fracture with bilateral sternochondral disjunction as well as lateral flail segments. A simple lateral fixation or Nuss procedure would not be adequate for chest wall stabilization. A modified Ravitch procedure, an operation typically reserved for complex or recurrent cases of pectus excavatum [9], was employed in this situation. The key steps in this procedure involve the creation of myocutaneous flaps, subperichondral cartilage resection, and transverse sternal osteotomy with fixation to bring the sternum to a more anterior position, as originally described by Ravitch in 1949 [10].

Our surgical approach modifies this classic technique, which we found useful for several reasons. The transverse inframammary incision with a midline cephalad extension allows adequate access to both the sternal fracture as well as the lateral flail segments, all of which required surgical fixation to restore chest wall stability. Our modification to the Ravitch procedure also involves both reduction and fixation of the sternochondral cartilage and repositioning of the sternal fragments, both of which had been forcefully displaced as a result of the blunt trauma. The reconstruction of the sternal fracture was achieved with an H-plate, providing 8 sites of bicortical fixation along the fracture. This method of fixation has been shown to provide superior stabilization in comparison to sternal wires, which are used in the classic operation [11, 12]. The myocutaneous flaps we created provide additional coverage to the implanted hardware and reinforcement of the area, to prevent a possible pulmonary hernia.

## Conclusions

In summary, the management of flail chest continues to evolve as scientific evidence and experience accumulates. For certain subgroups of patients, surgical stabilization has been shown to improve outcomes. Anterior flail segments are rare and often more complex in comparison to lateral segments and thus can present challenges to surgical fixation. We have used a modified Ravitch procedure to successfully repair a severe sternal displacement with an associated flail segment. This technique can be safely and effectively used to provide adequate chest wall stabilization in rare, complex anterior injuries to improve recovery of pulmonary function in patients’ refractory to non-operative management.
